# Prognostic and predictive significance of long interspersed nucleotide element-1 methylation in advanced-stage colorectal cancer

**DOI:** 10.1186/s12885-016-2984-8

**Published:** 2016-12-12

**Authors:** Mami Kaneko, Masanori Kotake, Hiroyuki Bando, Tetsuji Yamada, Hirofumi Takemura, Toshinari Minamoto

**Affiliations:** 1 Department of General and Cardiothoracic Surgery, Graduate School of Medical Science, Kanazawa University, 13-1 Takara-machi, Kanazawa, 920-8641 Japan; 2 Department of Gastrointestinal Surgery, Ishikawa Prefectural Central Hospital, Kanazawa, Japan; 3 Division of Translational and Clinical Oncology, Cancer Research Institute, Kanazawa University, Kanazawa, Japan

**Keywords:** LINE-1 elements, Oxaliplatin, Colorectal cancer, Prognosis, Metastasis

## Abstract

**Background:**

Hypomethylation of Long Interspersed Nucleotide Element-1 (LINE-1) is associated with worse prognosis in colorectal cancer (CRC). However, little is known about the relevance of this marker for the prognosis and response to chemotherapy of metastatic and recurrent (advanced-stage) CRC. Our aim was therefore to investigate whether tumor LINE-1 hypomethylation correlates with patient survival and with response to 5-fluorouracil (5-FU)/ oxaliplatin (FOLFOX) chemotherapy in advanced-stage CRC.

**Methods:**

The study included 40 CRC patients who developed metastasis or local recurrence after surgery and subsequently underwent FOLFOX therapy. Progression-free and overall survival were estimated using the Kaplan-Meier method. LINE-1 methylation levels in formalin-fixed and paraffin-embedded primary tumor tissues were measured by MethyLight assay and correlated with patient survival. In vitro analyses were also conducted with human colon cancer cell lines having different LINE-1 methylation levels to examine the effects of 5-FU and oxaliplatin on LINE-1 activity and DNA double-strand-breaks.

**Results:**

Patients with LINE-1 hypomethylation showed significantly worse progression-free (median: 6.6 *vs* 9.4 months; *P* = 0.02) and overall (median: 16.6 *vs* 23.2 months; *P* = 0.01) survival following chemotherapy compared to patients with high methylation. LINE-1 hypomethylation was an independent factor for poor prognosis (*P* = 0.018) and was associated with a trend for non-response to FOLFOX chemotherapy. In vitro analysis showed that oxaliplatin increased the LINE-1 score in LINE-1-expressing (hypomethylated) cancer cells, thereby enhancing and prolonging the effect of 5-FU against these cells. This finding supports the observed correlation between tumor LINE-1 methylation and response to chemotherapy in CRC patients.

**Conclusions:**

Tumor LINE-1 hypomethylation is an independent marker of poor prognosis in advanced-stage CRC and may also predict non-response to combination FOLFOX chemotherapy. Prospective studies are needed to optimize the measurement of tumor LINE-1 methylation and to confirm its clinical impact, particularly as a predictive marker.

**Electronic supplementary material:**

The online version of this article (doi:10.1186/s12885-016-2984-8) contains supplementary material, which is available to authorized users.

## Background

Colorectal cancer (CRC) is one of the most common cancer types worldwide and was responsible for an estimated 694,000 deaths in 2012 [1]. Although the incidence of CRC is increasing, mortality from CRC has decreased in many countries [1]. This trend is likely due to early diagnosis and the development of multidisciplinary treatments [2]. Chemotherapy with 5-fluorouracil (5-FU) has been the key drug for the last 50 years. Untreated patients with metastatic CRC have a median survival period of only 8 months, whereas this is prolonged to 16.2–19.5 months by treatment with 5-FU and leucovorin in combination with oxaliplatin or irinotecan [3, 4].

Aberrant hypermethylation of tumor DNA is thought to contribute to the development and progression of cancer, including CRC, by silencing the expression of tumor suppressor genes [5]. On the other hand, genome-wide hypomethylation of tumor DNA induces genomic instability by reactivating transposable DNA sequences and this change has also been associated with colorectal carcinogenesis [6–8]. Emerging evidence indicates that epigenetic mechanisms such as aberrant DNA methylation can trigger resistance to 5-FU, oxaliplatin and irinotecan in CRC [9]. Oxaliplatin exerts its cytotoxic effects via DNA damage and the arrest of nucleic acid synthesis [10]. The different mechanisms of resistance to oxaliplatin include histone methylation and various gene alterations. However, the most important mechanism appears to involve the DNA mismatch repair (MMR) and nucleotide excision repair (NER) systems [11, 12]. Although tumor DNA hypermethylation has been implicated in chemoresistance [13, 14], little is known about its relationship to oxaliplatin resistance.

Long interspersed nucleotide element-1 (LINE-1) is one of the retrotransposons that are distributed throughout the genome. LINE-1 is about 6 kb long, occupies approximately 18% of the genome and plays a role in regulating genomic structure and function [15, 16]. Its insertion into gene promoters and exons results in the functional disruption of these sequences, while its insertion into introns causes exon skipping, alternative splicing and transcriptional attenuation [17]. Because of the high frequency of LINE-1 in the genome, its hypomethylation provides an accurate representation of global DNA hypomethylation [8, 18]. Tumor LINE-1 hypomethylation has consistently been associated with worse survival in CRC patients [19–21]. We also reported earlier that LINE-1 hypomethylation was predictive of good response to oral fluoropyrimidines in the adjuvant setting [22] and that LINE-1 methylation levels in primary tumors correlated well with those of metastatic lesions from the same patient [23, 24]. Recently, the predictive and prognostic significance of LINE-1 hypomethylation has been studied by several independent groups in metastatic (Stage IV) CRC using large patient cohorts [25, 26]. Furthermore, the potential association of LINE-1 hypomethylation with therapeutic efficacy of FOLFOX (combined folate, 5-fluorouracil and oxaliplatin) regimen has also been reported [27]. However, so far there are no systemic studies on the prognostic and predictive significance of LINE-1 hypomethylation in metastatic CRC patients who undergo treatment with FOLFOX. The aim of the present study was therefore to determine whether the level of LINE-1 methylation in the primary tumor of advanced-stage CRC patients was associated with overall survival and with response to FOLFOX combination chemotherapy. We also investigated whether LINE-1 methylation levels in colon cancer cell lines were associated with sensitivity to 5-FU or oxaliplatin.

## Methods

### CRC patients and tissue samples

This study included 40 patients with CRC who underwent surgery between October 1999 and November 2010 at the Ishikawa Prefectural Central Hospital (clinical details shown in Table 1). Surgical specimens including primary tumor were fixed by neutralized formalin and embedded in paraffin for routine histopathology diagnosis. Between 2005 and 2011, patients who developed metastasis or unresectable local recurrence after surgery were treated with a FOLFOX regimen consisting of bolus intravenous injection with oxaliplatin (85 mg/m^2^), leucovorin (200 mg/m^2^) and 5-FU (400 mg/m^2^), followed by continuous infusion over 46 h with 5-FU (2,400 mg/m^2^) [28]. This regimen was repeated every 2 weeks until disease progression or the occurrence of intolerable toxicities. Response to the FOLFOX regimen was evaluated over 4 to 6 courses of treatment according to the Response Evaluation Criteria in Solid Tumors (RECIST version 1.1) [29]. None of the patients received 5-FU-based chemotherapy before primary surgery. Progression-free survival (PFS), overall survival (OS) and 5-year overall probability of survival (OPS) were all defined from the time of initiation of chemotherapy.

**Table 1:** Association between the tumor LINE-1 methylation levels and clinicopathological characteristics of the advanced-stage CRC patients

	*n*	LINE-1 methylation		*p*-value
Gender				
Male	26	46.8	(39.8–53.2)	0.738
Female	15	48.5	(43.6–53.0)	
Age (years)				
65≥	20	47.4	(43.1–51.7)	0.806
>65	21	47.4	(41.4–52.6)	
Sites of primary tumors				
Colon	25	47.2	(42.2–51.7)	0.517
Rectum	16	47.7	(43.0–54.2)	
Tumor histological types				
Well-differentiated	4	47.6	(45.6–52.9)	0.941
Moderately-differentiated	34	47.5	(42.5–53.2)	
Poorly-differentiated	3	46.7	(41.5–52.9)	
Status				
Advanced (stage IV)	18	45	(42.1–48.9)	0.113
Recurrence	23	49.3	(43.3–54.4)	
Distant metastasis				
One organ	29	48.4	(43.8–54.0)	0.185
Multiple organs	12	45	(40.7–51.4)	
Previous treatment with 5-FU				
Yes	22	48.8	(42.5–53.2)	0.429
No	19	45.8	(42.6–51.7)	
Number of previous regimens				
0	30	46.8	(43.5–52.3)	0.612
1	11	49.1	(40.1–55.8)	

LINE-1 methylation levels are shown as the median value (25th–75th percentile). Histological type of the primary tumor was classified into well-, moderately- and poorly-differentiated adenocarcinoma according to their grading. 5-FU, 5-fluorouracil; LINE-1, long interspersed nucleotide element-1; n, number of patients

Tumor tissue was identified in representative 10 μm-thick paraffin sections. Following deparaffinization the genomic DNA was isolated using QIAamp DNA mini kits (Qiagen, Hilden, Germany).

### Colon cancer cell lines

Colon cancer cell lines SW480, HCT116, Caco-2 and RKO were obtained from the American Type Culture Collection (Manassas, VA, USA). The cells were maintained at 37 °C with 5% CO_2_ in Dulbecco’s modified Eagle’s medium (DMEM) supplemented with 10% fetal bovine serum and antibiotics (100 units/mL penicillin G, 100 μg/mL streptomycin; Gibco, Grand Island, NY, USA).

### Drug treatment of cells

We determined the concentration of oxaliplatin (2 μM) for the treatment of colon cancer cells by reference to the reported IC_50_ (concentration resulting in 50% growth inhibition) [30]. The concentration of 5-FU (1 or 2 μM) was determined in our previous study [22]. Cells were cultured for 24 h and then treated with oxaliplatin for 2 h. Following replacement of the medium, cells were treated with 5-FU for 48 h or 120 h. The effects of drug treatment were examined by measuring LINE-1 activity and DNA double-strand breaks as described below.

### LINE-1 methylation analysis

Genomic DNA was extracted from formalin-fixed and paraffin-embedded (FFPE) tumor tissues and cultured cells and treated with bisulfite using the EpiTect Plus Bisulfite Conversion kit (QIAGEN). LINE-1 methylation in CRC tumor tissues was measured by using the MethyLight assay as described previously [31]. LINE-1 methylation in CRC cell lines was quantified using methylation-specific real-time PCR [32] with the ABI-PRISM 7900 Sequence Detection System (Applied Biosystems, Osaka, Japan) and Premix Ex Taq (TaKaRa Bio, Otsu, Japan). The primers and probes used for LINE-1 methylation analyses [23, 32] were shown in Additional file 1: Table S1 and Additional file 2: Figure S1. The level of LINE-1 methylation was expressed as a median value with 25th–75th percentile. LINE-1 methylation levels for the colon cancer cells used in this study were determined in our previous study [22].

### Formaldehyde-assisted isolation of regulatory elements (FAIRE)

FAIRE isolates DNA regions with active chromatin and therefore enriches in functional genomic elements such as active promoters and transcriptional start sites. This was performed as described earlier [33] to enrich and measure the active promoter of LINE-1 in colon cancer cells. It is reported that DNA without histone binding isolated by FAIRE is active in retrotransposons including LINE-1 [34]. Following treatment of cells with 1% formaldehyde for 5 min, glycine was added to a final concentration of 125 mM for 5 min. The cross-linked chromatin was sheared by sonication and this was followed by phenol-chloroform extraction. Covalently linked protein-DNA complexes were sequestered into the organic phase, leaving protein-free DNA fragments in the aqueous phase. The DNA was precipitated by incubation with sodium acetate, glycogen and ethanol at −20 °C overnight. The amount of LINE-1 was measured by quantitative real-time PCR as described above and the LINE-1 score was obtained using the ∆∆Ct method. Design of the primers used for the real-time PCR was shown in Additional file 1: Table S1 and Additional file 2: Figure S1.

### Measurement of phospho-histone H2A.X (γH2A.X) foci

Cells were plated onto coverslips for 24 h prior to treatment with drugs as described above. Following treatment, cells were fixed in 4% paraformaldehyde for 10 min at room temperature and permeabilized for 5 min in 0.2% Triton X-100. After blocking with 5% skim milk for 60 min, cells were incubated with antibody to γH2A.X (Millipore, Darmstadt, Germany) for 90 min at 37 °C. The cells were then incubated with secondary antibody (Cy^TM^3-conjugated AffiniPure F(ab’)_2_ Fragment Goat Anti-Mouse IgG(H + L), Molecular Probes, Eugene, Oregon, USA) for 60 min at 37 °C. Immunostained cells were observed by fluorescence microscopy in order to score γH2A.X foci.

### Statistical analysis

Mann–Whitney *U*-test, Kruskal-Wallis or Fisher’s exact test were used to compare LINE-1 methylation levels between variables. A receiver operating characteristic (ROC) curve was created to evaluate the threshold of LINE-1 methylation level. Kaplan-Meier analysis and the log-rank test were used to evaluate differences in survival between patient groups. The prognostic significance of multiple variables was evaluated using a Cox proportional hazard regression model. Scores for γH2A.X foci were compared by Mann–Whitney *U*-test. All P-values shown are two-tailed, with *P* < 0.05 taken as significant. All statistical analyses were performed with EZR (Saitama Medical Center, Jichi Medical University, Saitama, Japan), which is a graphical user interface for R (The R Foundation for Statistical Computing, Vienna, Austria, version 3.1.2). It is a modified version of R commander (version 2.1-5) designed to add statistical functions that are frequently used in biostatistics [35].

## Results

### Tumor LINE-1 hypomethylation correlated with poor response to FOLFOX and with worse survival of advanced-stage CRC patients

**Fig. 1:**
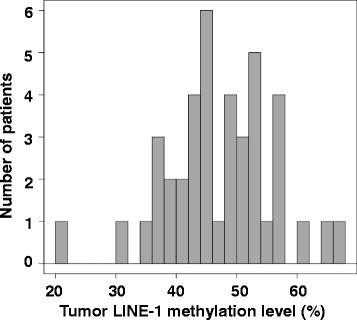
Distribution of LINE-1 methylation level in the primary tumors of advanced-stage CRC. The median level of LINE-1 methylation was 47.4%

**Fig. 2:**
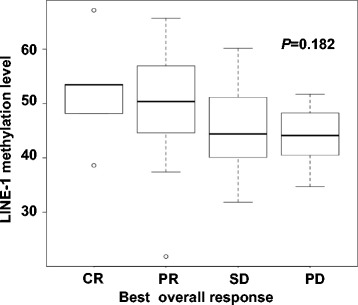
Tumor LINE-1 methylation levels in CRC patient groups classified according to their best overall response. LINE-1 methylation levels are shown as the median and 25th–75th percentile. CR, complete remission; PR, partial remission; SD, stable disease; PD, progressive disease

LINE-1 methylation (median; 25th–75th percentile) in the primary tumor of advanced-stage CRC patients (47.4%; 42.2–53.5%) (Fig. 1; Additional file 3: Figure S2A) was lower than in our previous study [22] of stage II and III CRC (84.7%; 27.8–94.0%). None of the patients in the present series was categorized as being in the high LINE-1 methylation (>84.3%) group defined in our previous study [22]. No significant associations were found between LINE-1 methylation and clinicopathological characteristics including age, sex, primary tumor site, metastatic sites and prior treatment with 5-FU (Table 1). Higher LINE-1 methylation levels were associated with a trend for better clinical response to FOLFOX chemotherapy (Fig. 2; Additional file 3: Figure S2B), however this did not reach statistical significance (*P* = 0.18).

**Fig. 3:**
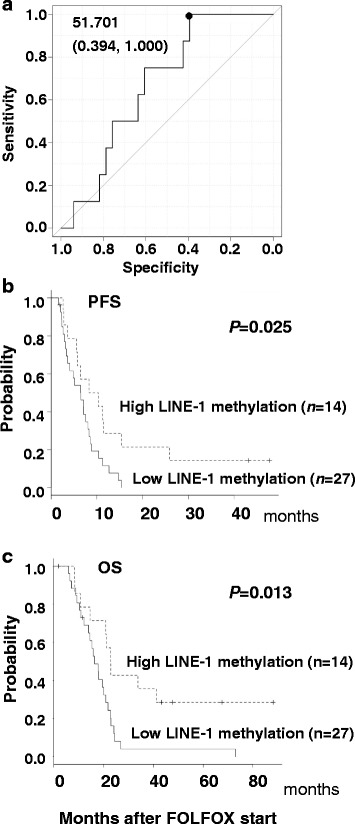
Comparison of tumor LINE-1 methylation levels with the survival of advanced-stage CRC patients. **a** ROC curve was constructed according to response (CR, PR and SD) or no response (PD) to the FOLFOX regimen. The cut-off value was determined as the point yielding the greatest sum of sensitivity and specificity for prediction of outcome. **b**, **c** Kaplan-Meier analysis of progression-free and overall survival of patient groups stratified according to their tumor LINE-1 methylation level. The log-rank test was used for each comparison and p-values are shown

An ROC curve was constructed to assess response (complete response, partial response and stable disease) or no response (progressive disease) to FOLFOX chemotherapy according to tumor LINE-1 methylation (Fig. 3a). The cutoff value for LINE-1 methylation level was 51.7%, as determined by the point yielding the greatest sum for sensitivity and specificity. The area under the curve (AUC) was 0.67 (95% CI: 0.49–0.85). Tumor LINE-1 methylation levels were thus classified as high (*n* = 14) or low (*n* = 27) according to this cutoff value. No significant differences in clinical and histopathological characteristics were apparent between the high and low LINE-1 methylation patient groups (Additional file 4: Table S2). However, the PFS, OS and 5-year OPS were significantly worse in the low compared to high methylation group (PFS: median 6.6 *vs.* 9.4 months, *P* = 0.02; OS: median 16.6 *vs.* 23.2 months, *P* = 0.01; 5-year OPS: 4.1% *vs.* 28.6%) (Fig. 3b, c). Multivariate analysis by COX proportional hazard model demonstrated that LINE-1 hypomethylation was an independent factor for poor prognosis (*P* = 0.018; Table 2, Additional file 5: Table S3, Additional file 6: Figure S3).

**Table 2:** Multivariate analysis for prognostic significance of clinicopathologic factors and tumor LINE-1 methylation in metastatic CRC

Variables	Hazard ratio	(95% CI)	*p*-value
Female	0.85	(0.38–1.91)	0.70
Older patients^a^	0.63	(0.28–1.41)	0.26
Rectum (vs. colon)	0.84	(0.38–1.85)	0.66
Well differentiation (vs. others)	0.95	(0.26–3.41)	0.94
Recurrence (vs. stage IV)	0.81	(0.31–2.14)	0.67
Multiple organs (vs. one organ) metastasis	0.85	(0.34–2.08)	0.72
Previous treatment with 5-FU	1.62	(0.55–4.77)	0.38
Second line (vs. first line) chemotherapy	1.08	(0.37–3.11)	0.89
LINE-1 hypomethylation ^b^	2.74	(1.19–6.29)	0.018

^a^Age: older (>65 y) versus younger (65 y ≥) patients

^b^The levels of tumor LINE-1 methylation were classified as hypomethylation versus hypermethylation based on the cutoff value (51.7%) determined by the ROC curve (Fig. 3a)

### Effects of oxaliplatin on LINE-1 activity and DNA damage in colon cancer cells

**Fig. 4:**
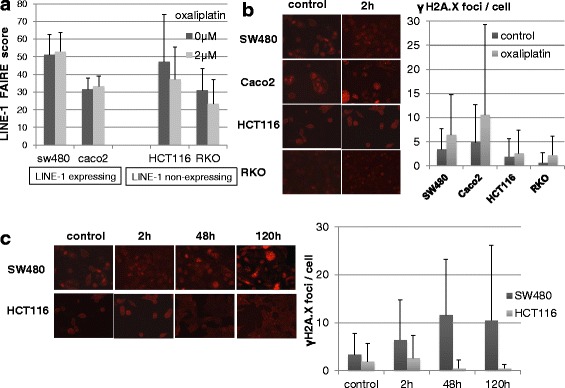
Effects of oxaliplatin on LINE-1 activity and γH2A.X foci in colon cancer cell lines. **a** Comparison of LINE-1 scores determined by FAIRE in LINE-1-expressing (SW480, Caco2) and non-expressing (HCT116, RKO) colon cancer cells that were treated with or without 2 μM oxaliplatin for 2 h. **b** Comparison of γH2A.X foci in colon cancer cells treated with or without 2 μM oxaliplatin for 2 h. **c** Comparison of γH2A.X foci between SW480 and HCT116 cells treated with or without 2 μM oxaliplatin for 2, 48 and 120 h, respectively

It was previously reported that SW480 and Caco2 cells show low LINE-1 methylation (55%) and express LINE-1, whereas HCT116 and RKO cells have higher LINE-1 methylation (65–90%) and express little or no LINE-1 [22, 36]. The effect of oxaliplatin on DNA stability in colon cancer cells with different levels of LINE-1 methylation was examined here using FAIRE. The baseline score for LINE-1 activity was higher in SW480 cells compared to HCT116, RKO and Caco2 cells, although the latter cell line also expressed LINE-1 (Fig. 4a; Additional file 7: Figure S4). Treatment of cells with 2 μM oxaliplatin for 2 h decreased the LINE-1 score in HCT116 and RKO, but not in SW480 and Caco2 cells. This suggests that oxaliplatin stabilizes the DNA of colon cancer cells with high LINE-1 methylation by attenuating LINE-1 transposition activity.

Next, the level of DNA damage in cells following treatment with oxaliplatin was evaluated by scoring the number of γH2A.X foci. This value reflects the extent of DNA double-strand breaks. In the untreated condition, γH2A.X foci (median; range) were more frequently (*P* < 0.01) detected in LINE-1-expressing SW480 (3.37; 0–20) and Caco2 cells (4.89; 0–40) than in LINE-1-non-expressing HCT116 (1.90; 0–20) and RKO cells (0.64; 0–12) (Fig. 4b). Treatment with 2 μM oxaliplatin for 2 h increased the number of foci in all cell lines. The effect of oxaliplatin on SW480 and HCT116 cells was monitored for 2 h, 48 h and 120 h. While the number of γH2A.X foci in SW480 cells increased progressively following treatment with oxaliplatin, the effect was transient in HCT116 (Fig. 4c). These results suggest the DNA in LINE-1-expressing cells such as SW480 is unstable and that treatment with oxaliplatin reinforces the DNA damage for an extended period over 120 h.

### Combined effect of oxaliplatin and 5-FU on colon cancer cells

**Fig. 5:**
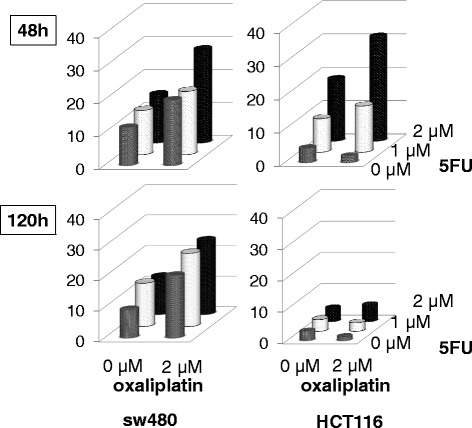
Combined effect of oxaliplatin and 5-FU treatment on LINE-1 expressing and non-expressing colon cancer cells. SW480 (LINE-1 expressing) and HCT116 (LINE-1 non-expressing) cells were treated with oxaliplatin (2 μM) alone, 5-FU (1 μM, 2 μM) alone, or a combination of both. For the combined treatment, the respective cells were treated sequentially with 2 μM oxaliplatin for 2 h and then with 5-FU (1 μM or 2 μM) for 48 h and 120 h. γH2A.X foci were measured following the respective treatments. Statistical differences between the data are shown in Additional file 4: Figure S2

The combined effect of oxaliplatin and 5-FU on DNA double-strand breaks was compared in LINE-1-expressing (SW480) and non-expressing (HCT116) cells (Fig. 5, Additional file 8: Figure S5). In SW480 cells, treatment with 2 μM 5-FU alone for 48 h increased the number of γH2A.X foci 1.28-fold compared to untreated cells. Pretreatment of SW480 cells with 2 μM oxaliplatin enhanced the effect of 5-FU between 1.49- and 1.92-fold and this effect persisted for up to 120 h. In HCT116 cells, treatment with 2 μM 5-FU alone for 48 h increased the number of γH2A.X foci 4.26-fold compared to untreated cells (*P* < 0.0005). Pretreatment of HCT116 cells with 2 μM oxaliplatin enhanced the effect of 5-FU by 7.16-fold (*P* < 0.0005), but no combined effect was found at 120 h after treatment. These results indicate that the effects of sequential treatment with oxaliplatin and 5-FU, which mimic the clinically used FOLFOX regimen, depend on the level of LINE-1 methylation in colon cancer cells.

## Discussion

Several previous studies have reported an association between tumor LINE-1 hypomethylation and worse survival of CRC patients from different clinical stages [19–21, 25–27]. We have also reported that 5-FU-based adjuvant chemotherapy appeared to benefit stage II and III CRC patients with low tumor LINE-1 methylation, but not those with high methylation [22]. In the present study we investigated LINE-1 methylation in the primary tumors of CRC patients with distant metastasis and/or recurrent tumors. We evaluated the prognostic relevance (PFS, OS, 5-year OPS) of LINE-1 methylation in patients who received chemotherapy with the combined oxaliplatin and 5-FU (FOLFOX) regimen. In agreement with the earlier studies cited above, tumor LINE-1 hypomethylation (i.e. below the cutoff value of 51.7% determined by ROC analysis) was associated with worse patient survival (Fig. 3b, c). In multivariate analysis, LINE-1 hypomethylation was shown to be an independent prognostic factor in advanced-stage CRC. The significant difference in survival between patients with high and low tumor LINE-1 methylation levels, together with the trend for inverse association between response to chemotherapy and LINE-1 methylation (Fig. 2), suggest this marker could help to select advanced-stage CRC patients who may benefit from FOLFOX.

We previously reported that stage II and III CRC patients with high LINE-1 methylation in their primary tumor did not show a survival benefit from adjuvant chemotherapy with oral 5-FU [22]. This apparent discrepancy with the current results from advanced stage CRC patients may be due to the different stages of the patient cohorts, the different chemotherapy regimen used, as well as the different cutoff values used to define high and low LINE-1 methylation levels. In previous studies, LINE-1 methylation levels were divided into the three categories of high, intermediate and low [22, 37, 38]. In our earlier study [22], patients defined as having “high” (≥84%) methylation showed favorable prognosis but little response to oral 5-FU chemotherapy. The “intermediate” (52–84%) and “low” (<52%) methylation tumor groups are susceptible to metastasis, while the former group is sensitive but the latter insensitive to 5-FU-based chemotherapy. According to this categorization and based on the cutoff value (51.7%) determined by the current ROC analysis (Fig. 3a), patients with high LINE-1 methylation as defined in the present study are included in the “intermediate” methylation group and there were no patients showing “high” methylation as defined above (≥84%). This could explain why patients with higher LINE-1 methylation (≥51.7%) showed better outcome following FOLFOX treatment compared to those with lower LINE-1 methylation (<51.7%). Consistent with previous studies [37, 38], patients with low LINE-1 methylation responded poorly to FOLFOX. Aside from the different CRC clinical stages and chemotherapy regimens, the difference in cutoff value for LINE-1 methylation (84.3% *vs.* 51.7%) between our previous [22] and present study does not allow direct comparison of the predictive value of LINE-1 methylation for response to 5-FU-based chemotherapy.

Previous [22, 37, 38] and present studies used DNA extracted from FFPE tumor tissues for bisulfite conversion and subsequent measurement of LINE-1 methylation. This may raise concern whether formalin fixation-associated DNA crosslinking and degradation will confound the quantitative PCR-based analysis of LINE-1 methylation. Therefore, this issue should be carefully addressed for clinical translation of LINE-1 methylation as a predictive biomarker in CRC.

To investigate putative mechanistic links between tumor LINE-1 methylation and therapeutic effects of the FOLFOX regimen, we examined human colon cancer cell lines with different LINE-1 methylation and expression levels. DNA double-strand breaks were induced by sequential combination treatment with oxaliplatin and 5-FU, thus mimicking the clinical FOLFOX regimen. The DNA damaging effect of this combined treatment was more marked and persistent in LINE-1-expressing SW480 cells (lower methylation) compared to HCT116 cells with no LINE-1 expression (higher methylation; Fig. 4c). This observation is consistent with previous reports that the DNA of LINE-1-expressing cancer cells is unstable due to its retrotransposition ability and is therefore inherently susceptible to cytotoxic insults [7, 8]. However, there is no direct evidence suggesting the correlation between LINE-1 expression and genomic instability. The different γH2A.X activity in different cell lines may be attributed to other sources such as intrinsic resistance (e.g., the ability of drug efflux) or the activities of DNA damage sensing and repair. The therapeutic effect observed in these colon cancer cells (Fig. 5) is inconsistent with the difference in outcome between advanced-stage CRC patients with high and low LINE-1 methylation levels treated with FOLFOX (Fig. 3). To address this discrepancy between clinical and in vitro findings, further investigations including verification of FFPE tissue samples as discussed above are needed on the expression of other known predictive biomarkers in experimental and clinical settings of combined oxaliplatin/5-FU regimens. These include the expression of thymidylate synthase [9] and dihydropyrimidine dehydrogenase [39] for 5-FU and excision-repair cross-complementing-1 for oxaliplatin [10].

Methylation of genes other than LINE-1, including *hMLH1*, *TFAP2E* and *SPARC*, have been associated with resistance to chemotherapy [14, 40–42]. Based on such findings, a phase I clinical trial was undertaken to test the safety and efficacy of azacitidine, a demethylating agent, in combination with oxaliplatin for patients with advanced cancers (including CRC) that had relapsed or were refractory to any platinum therapy [13].

## Conclusions

We have demonstrated that tumor LINE-1 hypomethylation is an independent marker of poor prognosis in advanced-stage CRC and may also be predictive of non-response to combination oxaliplatin/5-FU chemotherapy. Further prospective studies are required to establish whether tumor LINE-1 methylation can be a clinically useful prognostic and predictive biomarker for 5-FU-based chemotherapy in CRC patients. A critical issue will be to standardize the method for measuring tumor LINE-1 methylation level and for determining the cutoff value.

## Additional files

Additional file 1: Table S1. Primers and probes used for MethyLight assay, methylation-specific real-time PCR and FAIRE analysis of LINE-1. (DOCX 17 kb)
Additional file 2: Figure S1. The CpG sites in LINE-1 and design of primers used for methylation analysis. (PPTX 78 kb)
Additional file 3: Figure S2.
**(A)** Distribution of LINE-1 methylation level measured by using the primer set (2) (Additional file 1: Table S1; Additional file 2: Figure S1) in the primary tumors of advanced-stage CRC. **(B)** Tumor LINE-1 methylation levels in CRC patient groups classified according to their best overall response. LINE-1 methylation levels are measured by using the primer set (2) (Additional file 1: Table S1; Additional file 2: Figure S1) and shown as the median and 25th–75th percentile. CR, complete remission; PR, partial remission; SD, stable disease; PD, progressive disease. (PPTX 240 kb)
Additional file 4: Table S2. Comparison of the levels of tumor LINE-1 methylation with the clinical and histopathological characteristics of advanced-stage CRC patients. (DOCX 28 kb)
Additional file 5: Table S3. Multivariate analysis for the prognostic significance of clinicopathologic factors and tumor LINE-1 methylation in metastatic CRC. (DOCX 27 kb)
Additional file 6: Figure S3. Influence of clinical and histopathological parameters and of tumor LINE-1 methylation levels on the OS of patients was analyzed by Kaplan-Meier analysis. Levels of tumor LINE-1 methylation were classified as high or low based on the cutoff value (51.7%) determined by the ROC curve (Fig. 3a). (PPTX 664 kb)
Additional file 7: Figure S4. Comparison of LINE-1 scores determined by FAIRE in LINE-1-expressing (SW480, Caco2) and non-expressing (HCT116, RKO) colon cancer cells that were treated with or without 2 μM oxaliplatin for 2 hrs. The primer set (2) (Additional file 1: Table S1) was used for FAIRE analysis. (PPTX 38 kb)
Additional file 8: Figure S5. Statistical comparison of data shown in Fig. 5. Mann-Whitney U-test was used for each comparison and p-values are shown. (PPTX 161 kb)

## Abbreviations

5-FU: 5-fluorouracil
CRC: Colorectal cancer
FAIRE: Formaldehyde-assisted isolation of regulatory elements
FFPE: Formalin-fixed and paraffin-embedded
LINE-1: Long interspersed nucleotide element-1
OPS: Overall probability of survival
OS: Overall survival
PFS: Progression-free survival
ROC: Receiver operating characteristic
γH2A.X: Phosphorylated histone H2A.X
