# Supplementary material for: Prognostic and predictive significance of long interspersed nucleotide element-1 methylation in advanced-stage colorectal cancer
Source: BMC Cancer. 2016 Dec 12;16:945. doi: 10.1186/s12885-016-2984-8 (PMC5154037; doi:10.1186/s12885-016-2984-8)
Supplement: Additional file 1: Table S1. — Primers and probes used for MethyLight assay, methylation-specific real-time PCR and FAIRE analysis of LINE-1. (DOCX 17 kb) [file 12885_2016_2984_MOESM1_ESM.docx]

**Additional file 1: Table S1.** Primers and probes used for MethyLight assay, methylation-specific real-time PCR and FAIRE analysis of LINE1

| Analysis method | Sequence of primer and probe (5’ to 3’) |
| --- | --- |
| MethyLight assay (1) |  |
| forward | GGGAGTGTTAGATAGTGGG |
| reverse | ACCCAATTTTCCAAATACCATCT |
| MethyLight assay (2) |  |
| forward | GGGAGTGTTAGATAGTGGG |
| reverse | ACCCAATTTTCCAAATACATCCAT |
| MethyLight assay probes |  |
| unmethylated LINE-1 | FAM-CCTACTTCAACTCACACACAATAC-Eclipse Dark Quencher |
| methylated LINE-1 | Yakima yellow- CCTACTTCGACTCGCGCACGATAC-Eclipse Dark Quencher |
| Methylation-specific PCR |  |
| unmethylated LINE-1   forward  reverse | TGTGTGTGAGTTGAAGTAGGGT ACCCAATTTTCCAAATACAACCATCA |
| methylated LINE-1   forward  reverse | CGCGAGTCGAAGTAGGGC ACCCGATTTTCCAAATACGACCG |
| FAIRE analysis set (1) |  |
| forward | GCCAAGATGGCCGAATAGGA |
| reverse | TGTCTGGCACTCCCTAGTG |
| FAIRE analysis set (2) |  |
| forward | AACAGACCTGCAGCTGAGG |
| reverse | TGATGATGGTGATGTACAGATGG |

*The CpG sites in LINE-1 for methylation analysis were shown in Figure S1 (Additional file 2).
