# Supplementary material for: Prognostic and predictive significance of long interspersed nucleotide element-1 methylation in advanced-stage colorectal cancer
Source: BMC Cancer. 2016 Dec 12;16:945. doi: 10.1186/s12885-016-2984-8 (PMC5154037; doi:10.1186/s12885-016-2984-8)
Supplement: Additional file 2: Figure S1. — The CpG sites in LINE-1 and design of primers used for methylation analysis. (PPTX 78 kb) [file 12885_2016_2984_MOESM2_ESM.pptx]

## Slide 1
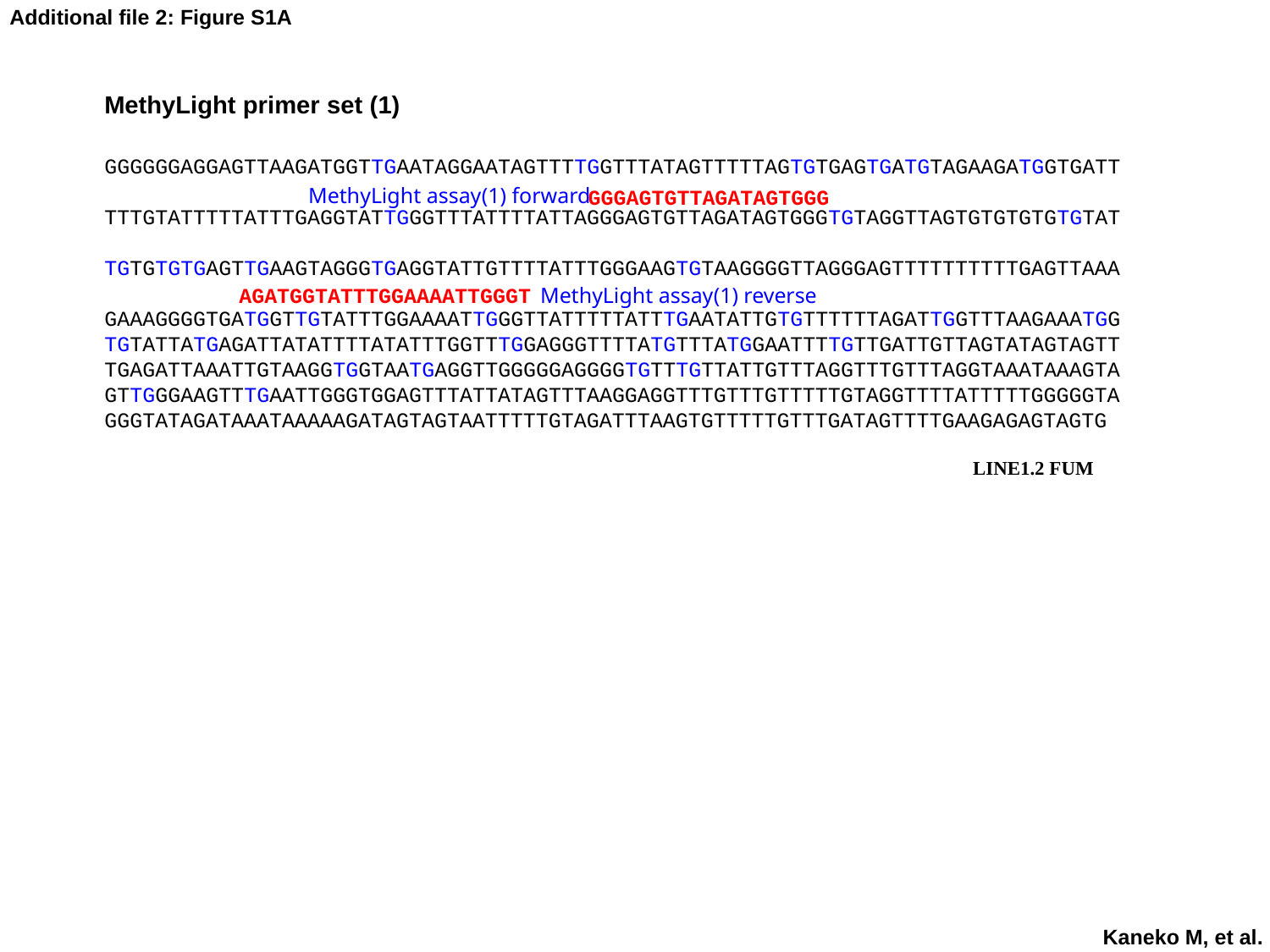

Additional file 2: Figure S1A
MethyLight primer set (1)
GGGGGGAGGAGTTAAGATGGTTGAATAGGAATAGTTTTGGTTTATAGTTTTTAGTGTGAGTGATGTAGAAGATGGTGATT
TTTGTATTTTTATTTGAGGTATTGGGTTTATTTTATTAGGGAGTGTTAGATAGTGGGTGTAGGTTAGTGTGTGTGTGTAT
TGTGTGTGAGTTGAAGTAGGGTGAGGTATTGTTTTATTTGGGAAGTGTAAGGGGTTAGGGAGTTTTTTTTTTGAGTTAAA
GAAAGGGGTGATGGTTGTATTTGGAAAATTGGGTTATTTTTATTTGAATATTGTGTTTTTTAGATTGGTTTAAGAAATGGTGTATTATGAGATTATATTTTATATTTGGTTTGGAGGGTTTTATGTTTATGGAATTTTGTTGATTGTTAGTATAGTAGTTTGAGATTAAATTGTAAGGTGGTAATGAGGTTGGGGGAGGGGTGTTTGTTATTGTTTAGGTTTGTTTAGGTAAATAAAGTAGTTGGGAAGTTTGAATTGGGTGGAGTTTATTATAGTTTAAGGAGGTTTGTTTGTTTTTGTAGGTTTTATTTTTGGGGGTAGGGTATAGATAAATAAAAAGATAGTAGTAATTTTTGTAGATTTAAGTGTTTTTGTTTGATAGTTTTGAAGAGAGTAGTG
MethyLight assay(1) forward
GGGAGTGTTAGATAGTGGG
AGATGGTATTTGGAAAATTGGGT
MethyLight assay(1) reverse
LINE1.2 FUM
Kaneko M, et al.

## Slide 2
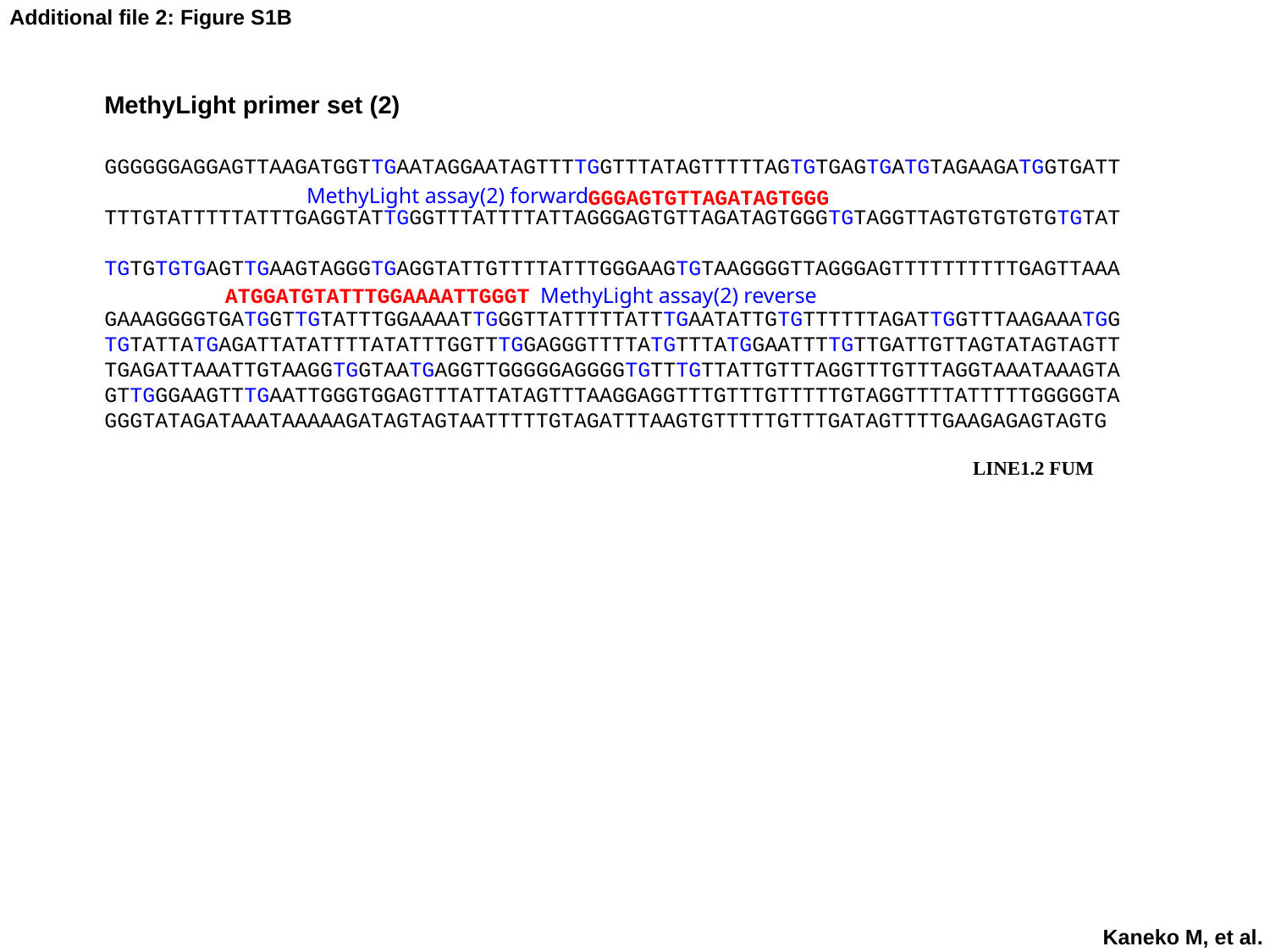

Additional file 2: Figure S1B
MethyLight primer set (2)
GGGGGGAGGAGTTAAGATGGTTGAATAGGAATAGTTTTGGTTTATAGTTTTTAGTGTGAGTGATGTAGAAGATGGTGATT
TTTGTATTTTTATTTGAGGTATTGGGTTTATTTTATTAGGGAGTGTTAGATAGTGGGTGTAGGTTAGTGTGTGTGTGTAT
TGTGTGTGAGTTGAAGTAGGGTGAGGTATTGTTTTATTTGGGAAGTGTAAGGGGTTAGGGAGTTTTTTTTTTGAGTTAAA
GAAAGGGGTGATGGTTGTATTTGGAAAATTGGGTTATTTTTATTTGAATATTGTGTTTTTTAGATTGGTTTAAGAAATGGTGTATTATGAGATTATATTTTATATTTGGTTTGGAGGGTTTTATGTTTATGGAATTTTGTTGATTGTTAGTATAGTAGTTTGAGATTAAATTGTAAGGTGGTAATGAGGTTGGGGGAGGGGTGTTTGTTATTGTTTAGGTTTGTTTAGGTAAATAAAGTAGTTGGGAAGTTTGAATTGGGTGGAGTTTATTATAGTTTAAGGAGGTTTGTTTGTTTTTGTAGGTTTTATTTTTGGGGGTAGGGTATAGATAAATAAAAAGATAGTAGTAATTTTTGTAGATTTAAGTGTTTTTGTTTGATAGTTTTGAAGAGAGTAGTG
MethyLight assay(2) forward
GGGAGTGTTAGATAGTGGG
ATGGATGTATTTGGAAAATTGGGT
MethyLight assay(2) reverse
LINE1.2 FUM
Kaneko M, et al.

## Slide 3
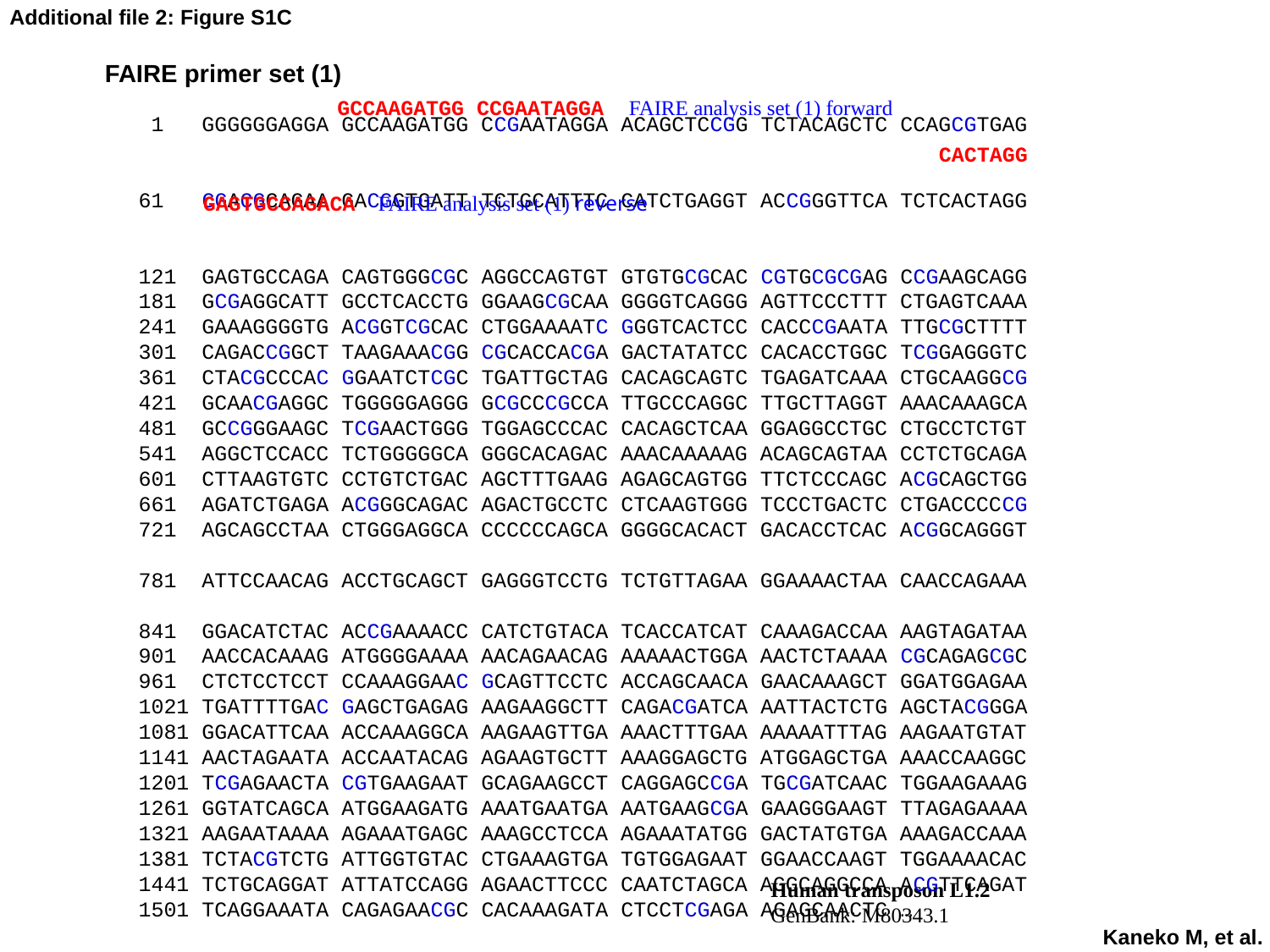

Additional file 2: Figure S1C
FAIRE primer set (1)
GCCAAGATGG CCGAATAGGA
FAIRE analysis set (1) forward
 1 GGGGGGAGGA GCCAAGATGG CCGAATAGGA ACAGCTCCGG TCTACAGCTC CCAGCGTGAG
61 CGACGCAGAA GACGGTGATT TCTGCATTTC CATCTGAGGT ACCGGGTTCA TCTCACTAGG
121 GAGTGCCAGA CAGTGGGCGC AGGCCAGTGT GTGTGCGCAC CGTGCGCGAG CCGAAGCAGG 181 GCGAGGCATT GCCTCACCTG GGAAGCGCAA GGGGTCAGGG AGTTCCCTTT CTGAGTCAAA 241 GAAAGGGGTG ACGGTCGCAC CTGGAAAATC GGGTCACTCC CACCCGAATA TTGCGCTTTT 301 CAGACCGGCT TAAGAAACGG CGCACCACGA GACTATATCC CACACCTGGC TCGGAGGGTC 361 CTACGCCCAC GGAATCTCGC TGATTGCTAG CACAGCAGTC TGAGATCAAA CTGCAAGGCG 421 GCAACGAGGC TGGGGGAGGG GCGCCCGCCA TTGCCCAGGC TTGCTTAGGT AAACAAAGCA 481 GCCGGGAAGC TCGAACTGGG TGGAGCCCAC CACAGCTCAA GGAGGCCTGC CTGCCTCTGT 541 AGGCTCCACC TCTGGGGGCA GGGCACAGAC AAACAAAAAG ACAGCAGTAA CCTCTGCAGA 601 CTTAAGTGTC CCTGTCTGAC AGCTTTGAAG AGAGCAGTGG TTCTCCCAGC ACGCAGCTGG 661 AGATCTGAGA ACGGGCAGAC AGACTGCCTC CTCAAGTGGG TCCCTGACTC CTGACCCCCG 721 AGCAGCCTAA CTGGGAGGCA CCCCCCAGCA GGGGCACACT GACACCTCAC ACGGCAGGGT
781 ATTCCAACAG ACCTGCAGCT GAGGGTCCTG TCTGTTAGAA GGAAAACTAA CAACCAGAAA
841 GGACATCTAC ACCGAAAACC CATCTGTACA TCACCATCAT CAAAGACCAA AAGTAGATAA 901 AACCACAAAG ATGGGGAAAA AACAGAACAG AAAAACTGGA AACTCTAAAA CGCAGAGCGC 961 CTCTCCTCCT CCAAAGGAAC GCAGTTCCTC ACCAGCAACA GAACAAAGCT GGATGGAGAA 1021 TGATTTTGAC GAGCTGAGAG AAGAAGGCTT CAGACGATCA AATTACTCTG AGCTACGGGA 1081 GGACATTCAA ACCAAAGGCA AAGAAGTTGA AAACTTTGAA AAAAATTTAG AAGAATGTAT 1141 AACTAGAATA ACCAATACAG AGAAGTGCTT AAAGGAGCTG ATGGAGCTGA AAACCAAGGC 1201 TCGAGAACTA CGTGAAGAAT GCAGAAGCCT CAGGAGCCGA TGCGATCAAC TGGAAGAAAG 1261 GGTATCAGCA ATGGAAGATG AAATGAATGA AATGAAGCGA GAAGGGAAGT TTAGAGAAAA 1321 AAGAATAAAA AGAAATGAGC AAAGCCTCCA AGAAATATGG GACTATGTGA AAAGACCAAA 1381 TCTACGTCTG ATTGGTGTAC CTGAAAGTGA TGTGGAGAAT GGAACCAAGT TGGAAAACAC 1441 TCTGCAGGAT ATTATCCAGG AGAACTTCCC CAATCTAGCA AGGCAGGCCA ACGTTCAGAT 1501 TCAGGAAATA CAGAGAACGC CACAAAGATA CTCCTCGAGA AGAGCAACTC …
CACTAGG
GAGTGCCAGACA
FAIRE analysis set (1) reverse
Human transposon L1.2
GenBank: M80343.1
Kaneko M, et al.

## Slide 4
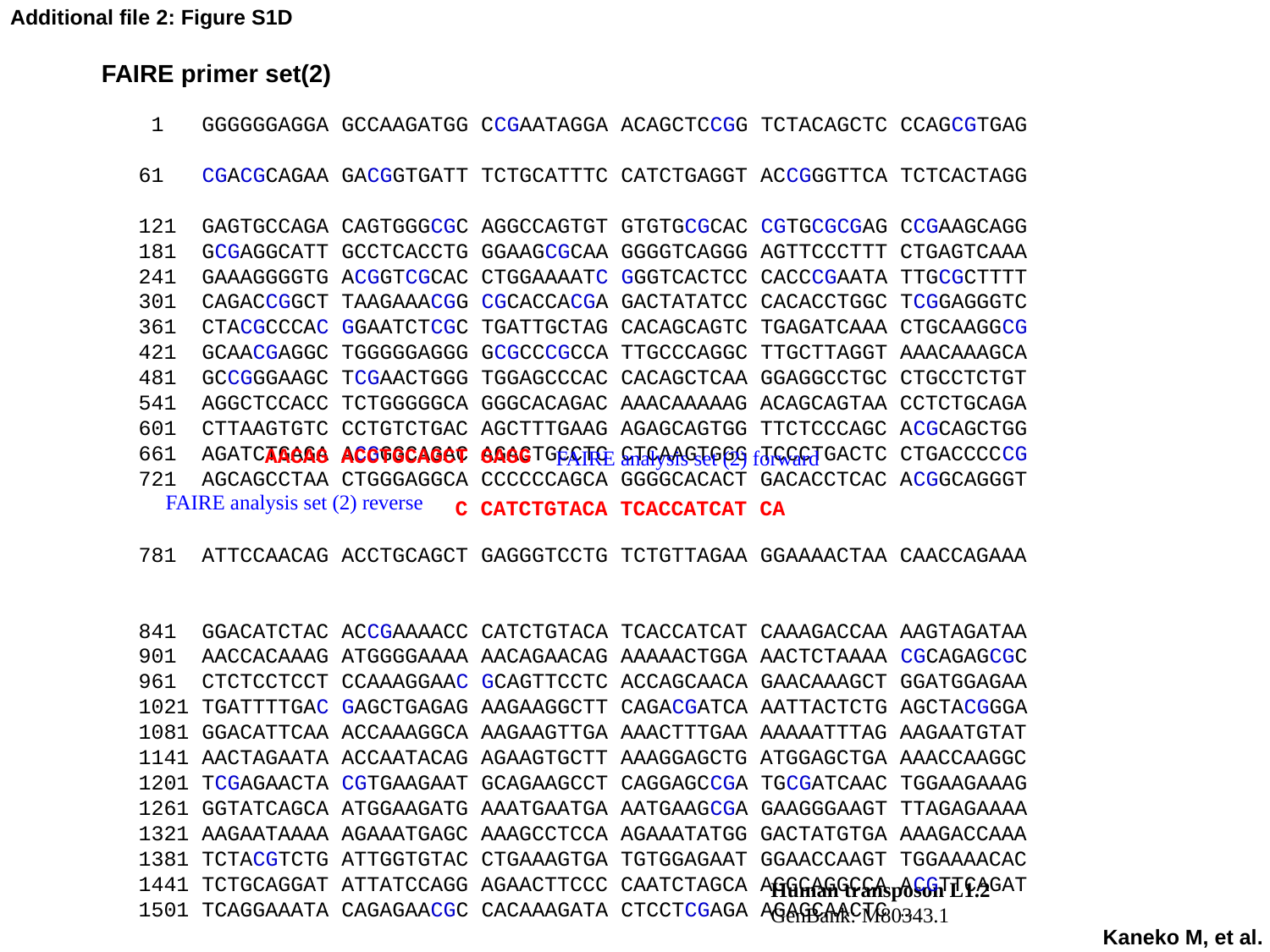

Additional file 2: Figure S1D
FAIRE primer set(2)
 1 GGGGGGAGGA GCCAAGATGG CCGAATAGGA ACAGCTCCGG TCTACAGCTC CCAGCGTGAG
61 CGACGCAGAA GACGGTGATT TCTGCATTTC CATCTGAGGT ACCGGGTTCA TCTCACTAGG
121 GAGTGCCAGA CAGTGGGCGC AGGCCAGTGT GTGTGCGCAC CGTGCGCGAG CCGAAGCAGG 181 GCGAGGCATT GCCTCACCTG GGAAGCGCAA GGGGTCAGGG AGTTCCCTTT CTGAGTCAAA 241 GAAAGGGGTG ACGGTCGCAC CTGGAAAATC GGGTCACTCC CACCCGAATA TTGCGCTTTT 301 CAGACCGGCT TAAGAAACGG CGCACCACGA GACTATATCC CACACCTGGC TCGGAGGGTC 361 CTACGCCCAC GGAATCTCGC TGATTGCTAG CACAGCAGTC TGAGATCAAA CTGCAAGGCG 421 GCAACGAGGC TGGGGGAGGG GCGCCCGCCA TTGCCCAGGC TTGCTTAGGT AAACAAAGCA 481 GCCGGGAAGC TCGAACTGGG TGGAGCCCAC CACAGCTCAA GGAGGCCTGC CTGCCTCTGT 541 AGGCTCCACC TCTGGGGGCA GGGCACAGAC AAACAAAAAG ACAGCAGTAA CCTCTGCAGA 601 CTTAAGTGTC CCTGTCTGAC AGCTTTGAAG AGAGCAGTGG TTCTCCCAGC ACGCAGCTGG 661 AGATCTGAGA ACGGGCAGAC AGACTGCCTC CTCAAGTGGG TCCCTGACTC CTGACCCCCG 721 AGCAGCCTAA CTGGGAGGCA CCCCCCAGCA GGGGCACACT GACACCTCAC ACGGCAGGGT
781 ATTCCAACAG ACCTGCAGCT GAGGGTCCTG TCTGTTAGAA GGAAAACTAA CAACCAGAAA
841 GGACATCTAC ACCGAAAACC CATCTGTACA TCACCATCAT CAAAGACCAA AAGTAGATAA 901 AACCACAAAG ATGGGGAAAA AACAGAACAG AAAAACTGGA AACTCTAAAA CGCAGAGCGC 961 CTCTCCTCCT CCAAAGGAAC GCAGTTCCTC ACCAGCAACA GAACAAAGCT GGATGGAGAA 1021 TGATTTTGAC GAGCTGAGAG AAGAAGGCTT CAGACGATCA AATTACTCTG AGCTACGGGA 1081 GGACATTCAA ACCAAAGGCA AAGAAGTTGA AAACTTTGAA AAAAATTTAG AAGAATGTAT 1141 AACTAGAATA ACCAATACAG AGAAGTGCTT AAAGGAGCTG ATGGAGCTGA AAACCAAGGC 1201 TCGAGAACTA CGTGAAGAAT GCAGAAGCCT CAGGAGCCGA TGCGATCAAC TGGAAGAAAG 1261 GGTATCAGCA ATGGAAGATG AAATGAATGA AATGAAGCGA GAAGGGAAGT TTAGAGAAAA 1321 AAGAATAAAA AGAAATGAGC AAAGCCTCCA AGAAATATGG GACTATGTGA AAAGACCAAA 1381 TCTACGTCTG ATTGGTGTAC CTGAAAGTGA TGTGGAGAAT GGAACCAAGT TGGAAAACAC 1441 TCTGCAGGAT ATTATCCAGG AGAACTTCCC CAATCTAGCA AGGCAGGCCA ACGTTCAGAT 1501 TCAGGAAATA CAGAGAACGC CACAAAGATA CTCCTCGAGA AGAGCAACTC …
Aacag acctgcagct gagg
FAIRE analysis set (2) forward
FAIRE analysis set (2) reverse
C catctgtaca tcacCatcat ca
Human transposon L1.2
GenBank: M80343.1
Kaneko M, et al.
