# Supplementary material for: Prognostic and predictive significance of long interspersed nucleotide element-1 methylation in advanced-stage colorectal cancer
Source: BMC Cancer. 2016 Dec 12;16:945. doi: 10.1186/s12885-016-2984-8 (PMC5154037; doi:10.1186/s12885-016-2984-8)
Supplement: Additional file 3: Figure S2. — (A) Distribution of LINE-1 methylation level measured by using the primer set (2) (Additional file 1: Table S1; Additional file 2: Figure S1) in the primary tumors of advanced-stage CRC. (B) Tumor LINE-1 methylation levels in CRC patient groups classified according to their best overall response. LINE-1 methylation levels are measured by using the primer set (2) (Additional file 1: Table S1; Additional file 2: Figure S1) and shown as the median and 25th–75th percentile. CR, complete remission; PR, partial remission; SD, stable disease; PD, progressive disease. (PPTX 240 kb) [file 12885_2016_2984_MOESM3_ESM.pptx]

## Slide 1
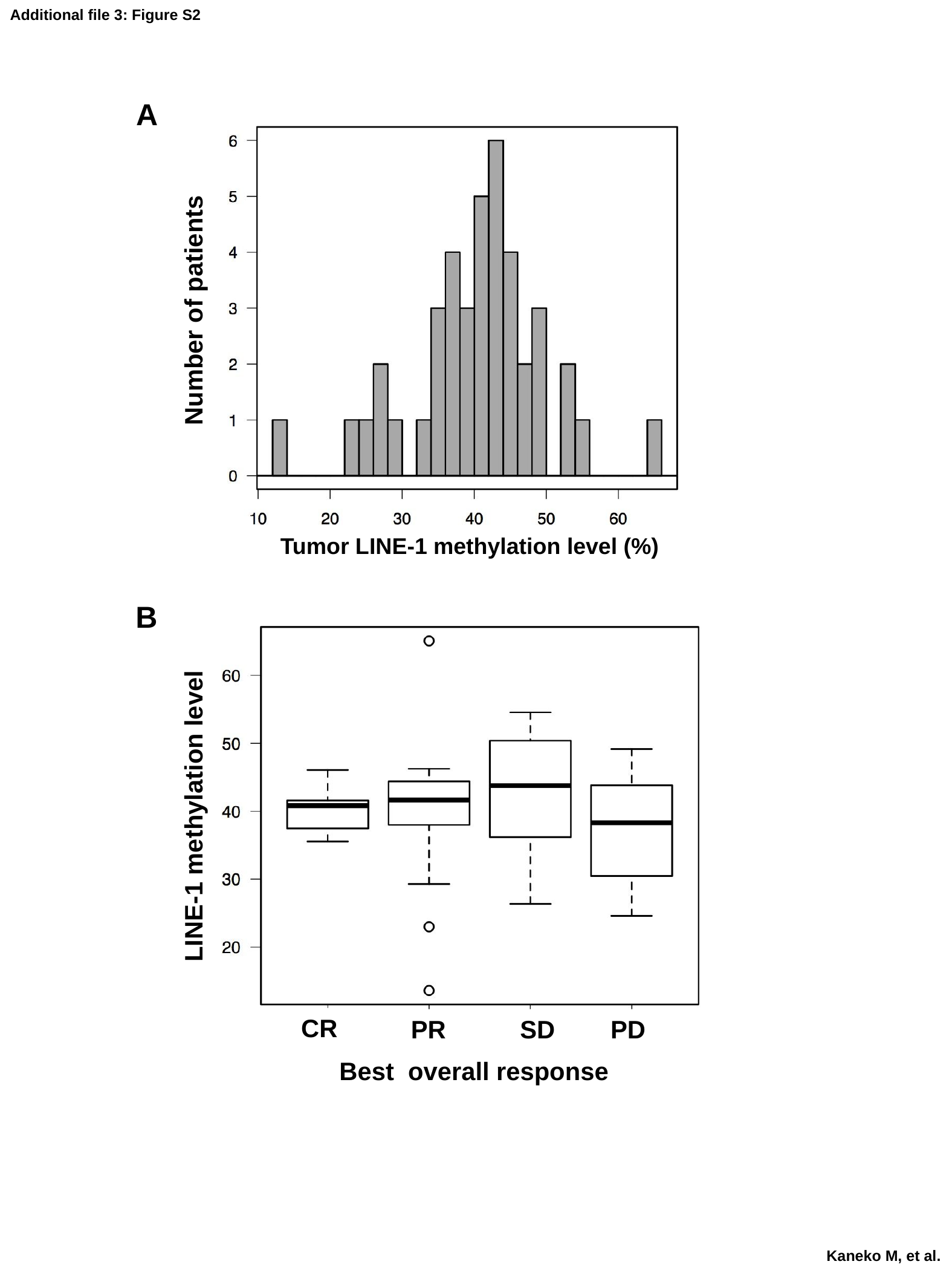

Additional file 3: Figure S2
Number of patients
Tumor LINE-1 methylation level (%)
A
LINE-1 methylation level
CR
PR
SD
PD
Best overall response
B
Kaneko M, et al.
