# Supplementary material for: Prognostic and predictive significance of long interspersed nucleotide element-1 methylation in advanced-stage colorectal cancer
Source: BMC Cancer. 2016 Dec 12;16:945. doi: 10.1186/s12885-016-2984-8 (PMC5154037; doi:10.1186/s12885-016-2984-8)
Supplement: Additional file 4: Table S2. — Comparison of the levels of tumor LINE-1 methylation with the clinical and histopathological characteristics of advanced-stage CRC patients. (DOCX 28 kb) [file 12885_2016_2984_MOESM4_ESM.docx]

**Additional file 4: Table S2.** Comparison of tumor LINE-1 methylation levels with clinical and histopathological characteristics of advanced-stage CRC patients.

|  |  | LINE-1 methylation  Low High  (n=27)　 (n=14) | | p-value |
| --- | --- | --- | --- | --- |
| Gender | |  |  |  |
|  | male | 17 | 9 | 1 |
|  | female | 10 | 5 |  |
| Age (years) | |  |  |  |
|  | 65 ≥ | 14 | 6 | 0.744 |
|  | > 65 | 13 | 8 |  |
| Sites of primary tumor | |  |  |  |
|  | colon | 18 | 7 | 0.332 |
|  | rectum | 9 | 7 |  |
| Tumor histological types | |  |  |  |
|  | well-differentiated | 2 | 2 | 0.596 |
|  | others | 25 | 12 |  |
| Status | |  |  |  |
|  | advanced (stage IV) | 15 | 3 | 0.0505 |
|  | recurrence | 12 | 11 |  |
| Distant metastasis | |  |  |  |
|  | one organ | 18 | 11 | 0.494 |
|  | multiple organs | 9 | 3 |  |
| Previous treatment with 5-FU | |  |  |  |
|  | yes | 13 | 9 | 0.51 |
|  | no | 14 | 5 |  |
| Number of previous regimens | |  |  |  |
|  | 0 | 21 | 9 | 0.463 |
|  | 1 | 6 | 5 |  |

The levels of tumor LINE-1 methylation were classified as high and low based on the cutoff value (51.7%) determined by the ROC curve (Figure 3A). Histological types of CRC were classified into well-differentiated adenocarcinoma and others according to their grading. LINE-1, long interspersed nucleotide element-1; n, number of patients. P-values were obtained by Fisher’s exact test.
