# Supplementary material for: Prognostic and predictive significance of long interspersed nucleotide element-1 methylation in advanced-stage colorectal cancer
Source: BMC Cancer. 2016 Dec 12;16:945. doi: 10.1186/s12885-016-2984-8 (PMC5154037; doi:10.1186/s12885-016-2984-8)
Supplement: Additional file 5: Table S3. — Multivariate analysis for the prognostic significance of clinicopathologic factors and tumor LINE-1 methylation in metastatic CRC. (DOCX 27 kb) [file 12885_2016_2984_MOESM5_ESM.docx]

**Additional file 5: Table S3.** Multivariate analysis for the prognostic significance of clinicopathologic factors and LINE-1 methylation in metastatic CRC

| Variable |  | n | Hazard ratio | (95% CI) | p-value |
| --- | --- | --- | --- | --- | --- |
| Gender | male | 26 | 1.00 |  |  |
|  | female | 15 | 0.85 | (0.38 - 1.91) | 0.70 |
| Age | 65≥ | 20 | 1.00 |  |  |
|  | >65 | 21 | 0.63 | (0.28 - 1.41) | 0.26 |
| Site | colon | 25 | 1.00 |  |  |
|  | rectum | 16 | 0.84 | (0.38 - 1.85) | 0.66 |
| Histological type of tumor | others | 37 | 1.00 |  |  |
|  | well | 4 | 0.95 | (0.26 - 3.41) | 0.94 |
| Status | Stage IV | 18 | 1.00 |  |  |
|  | recurrence | 23 | 0.81 | (0.31 - 2.14) | 0.67 |
| Distant metastasis | One organ | 29 | 1.00 |  |  |
|  | Multiple organs | 12 | 0.85 | (0.34 - 2.08) | 0.72 |
| Previous treatment with 5-FU | no | 19 | 1.00 |  |  |
|  | yes | 22 | 1.62 | (0.55 - 4.77) | 0.38 |
| Chemotherapy | First line | 30 | 1.00 |  |  |
|  | Second line | 11 | 1.08 | (0.37 - 3.11) | 0.89 |
| LINE-1 methylation† | High | 14 | 1.00 |  |  |
|  | Low | 27 | 2.74 | (1.19 - 6.29) | 0.018 |

†The levels of tumor LINE-1 methylation were classified as high versus low based on the cutoff value (51.7%) determined by the ROC curve (Figure 3A). LINE-1, long interspersed nucleotide element-1; n, number of patients.
