# Supplementary material for: Prognostic and predictive significance of long interspersed nucleotide element-1 methylation in advanced-stage colorectal cancer
Source: BMC Cancer. 2016 Dec 12;16:945. doi: 10.1186/s12885-016-2984-8 (PMC5154037; doi:10.1186/s12885-016-2984-8)
Supplement: Additional file 6: Figure S3. — Influence of clinical and histopathological parameters and of tumor LINE-1 methylation levels on the OS of patients was analyzed by Kaplan-Meier analysis. Levels of tumor LINE-1 methylation were classified as high or low based on the cutoff value (51.7%) determined by the ROC curve (Fig. 3a). (PPTX 664 kb) [file 12885_2016_2984_MOESM6_ESM.pptx]

## Slide 1
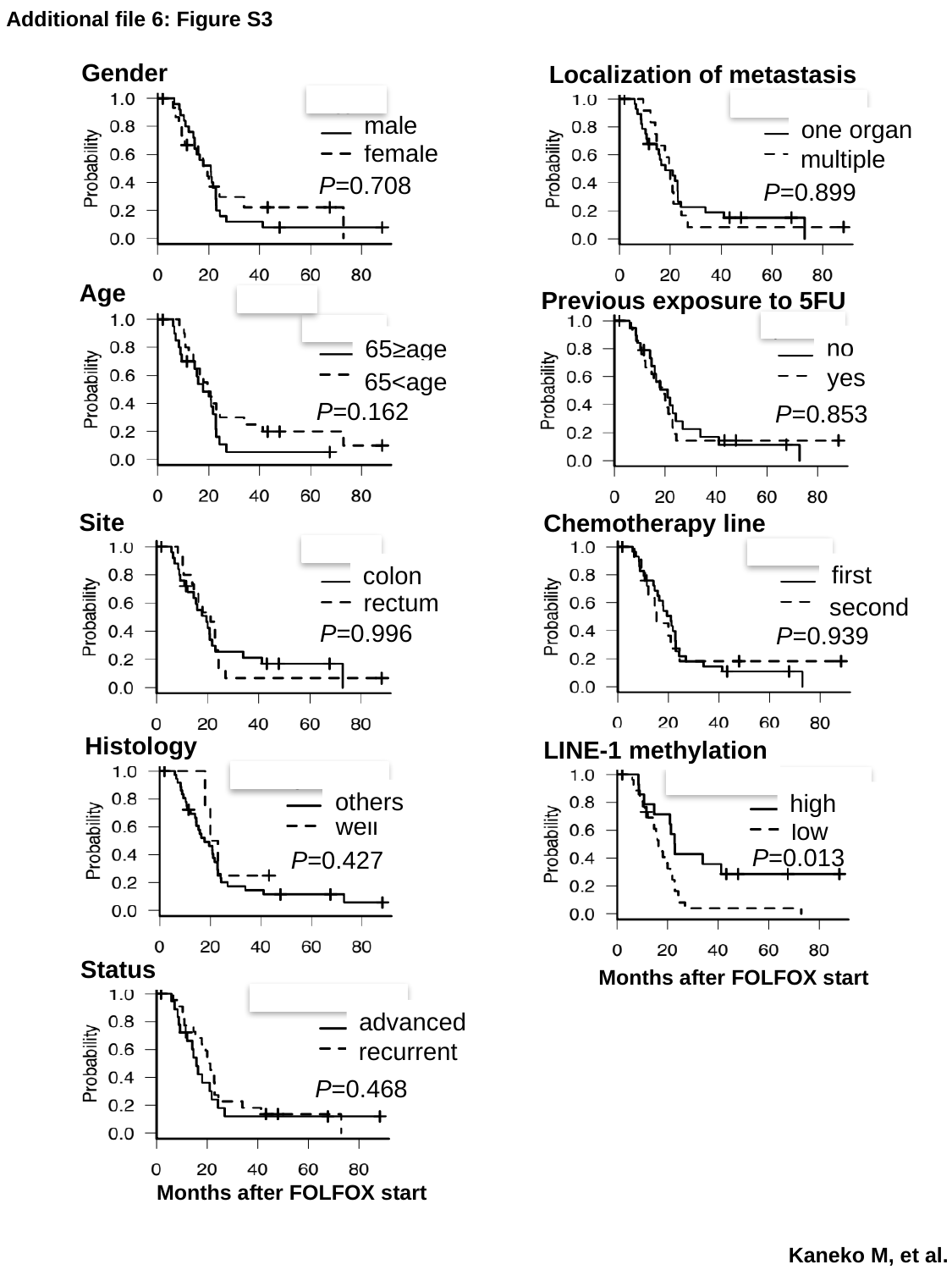

Additional file 6: Figure S3
Gender
male
female
P=0.708
Localization of metastasis
one organ
multiple
P=0.899
Age
65≥age
65<age
Previous exposure to 5FU
no
yes
P=0.162
P=0.853
Site
colon
rectum
P=0.996
Chemotherapy line
first
second
P=0.939
Histology
LINE-1 methylation
high
low
P=0.013
Months after FOLFOX start
others
well
P=0.427
Status
advanced
recurrent
P=0.468
Months after FOLFOX start
Kaneko M, et al.
