# Supplementary material for: Prognostic and predictive significance of long interspersed nucleotide element-1 methylation in advanced-stage colorectal cancer
Source: BMC Cancer. 2016 Dec 12;16:945. doi: 10.1186/s12885-016-2984-8 (PMC5154037; doi:10.1186/s12885-016-2984-8)
Supplement: Additional file 7: Figure S4. — Comparison of LINE-1 scores determined by FAIRE in LINE-1-expressing (SW480, Caco2) and non-expressing (HCT116, RKO) colon cancer cells that were treated with or without 2 μM oxaliplatin for 2 hrs. The primer set (2) (Additional file 1: Table S1) was used for FAIRE analysis. (PPTX 38 kb) [file 12885_2016_2984_MOESM7_ESM.pptx]

## Slide 1
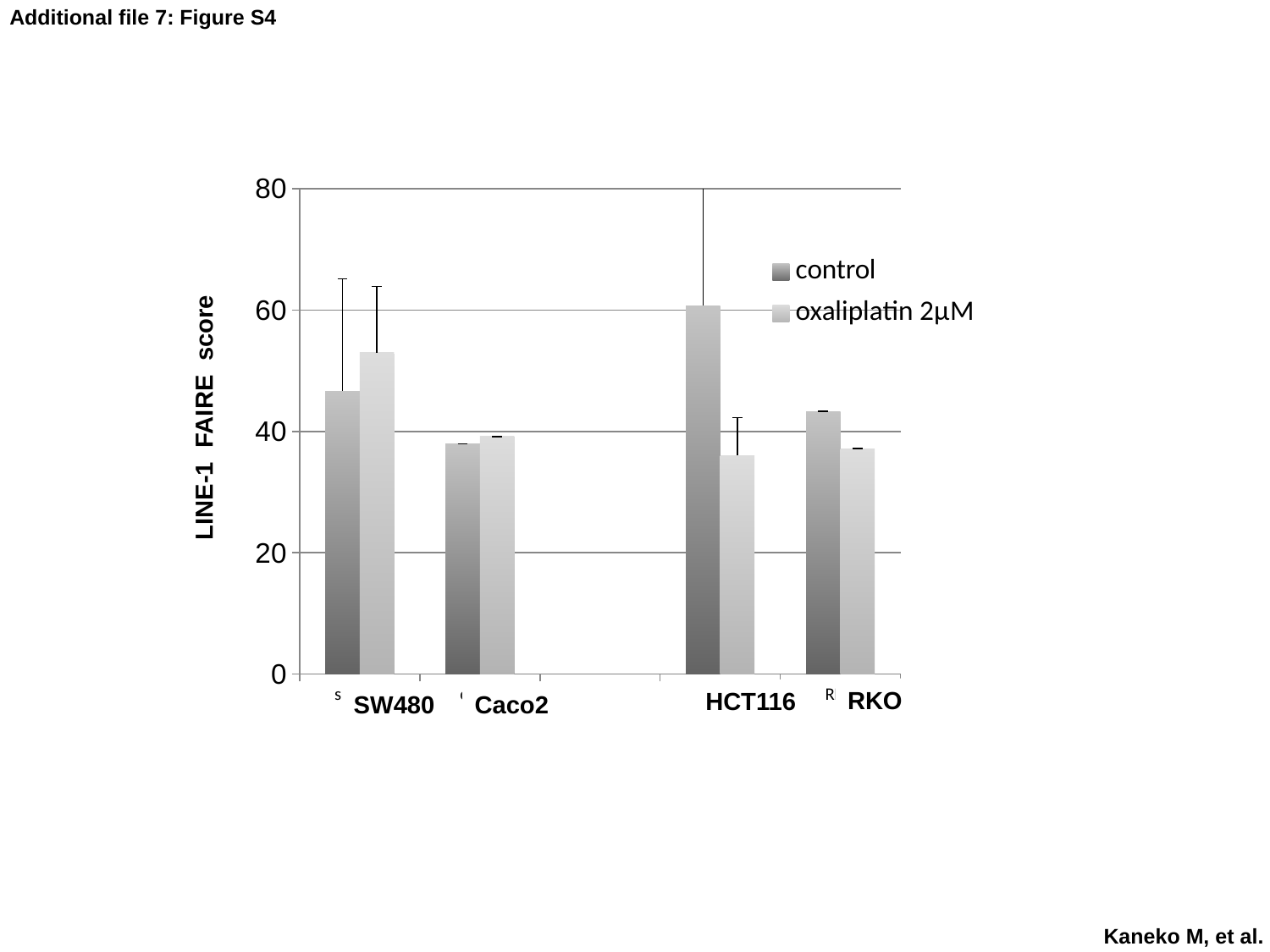

Additional file 7: Figure S4
### Chart
| Category | control | oxaliplatin 2μM |
|---|---|---|
| sw480 | 46.66 | 53.0 |
| caco2 | 37.94 | 39.14 |
| | None | None |
| HCT116 | 60.72 | 36.0 |
| RKO | 43.28 | 37.16 |LINE-1 FAIRE score
RKO
HCT116
SW480
Caco2
Kaneko M, et al.
