# Supplementary material for: Prognostic and predictive significance of long interspersed nucleotide element-1 methylation in advanced-stage colorectal cancer
Source: BMC Cancer. 2016 Dec 12;16:945. doi: 10.1186/s12885-016-2984-8 (PMC5154037; doi:10.1186/s12885-016-2984-8)
Supplement: Additional file 8: Figure S5. — Statistical comparison of data shown in Fig. 5. Mann-Whitney U-test was used for each comparison and p-values are shown. (PPTX 161 kb) [file 12885_2016_2984_MOESM8_ESM.pptx]

## Slide 1
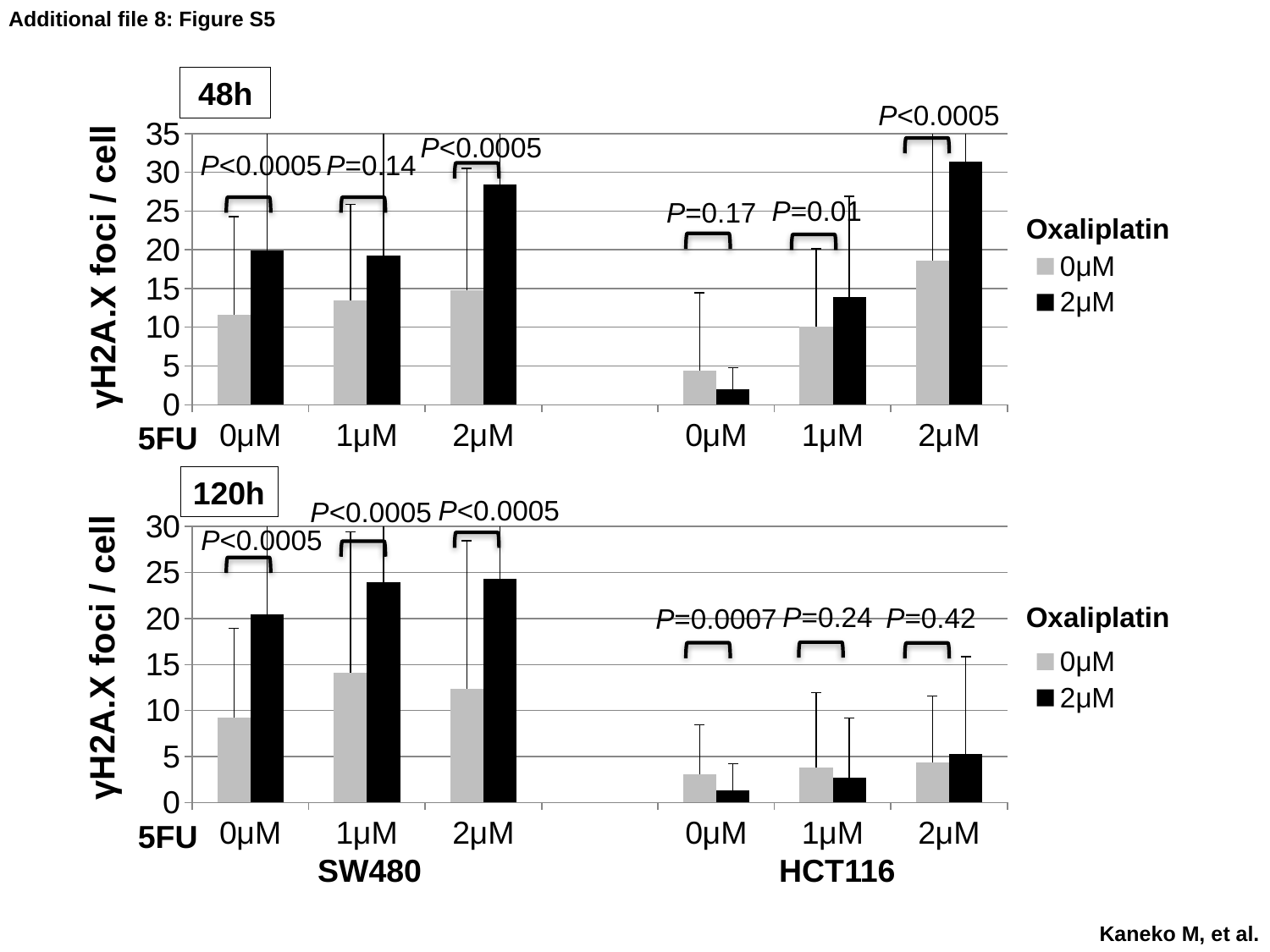

Additional file 8: Figure S5
48h
P<0.0005
### Chart
| Category | 0μM | 2μM |
|---|---|---|
| 0μM | 11.616 | 19.925 |
| 1μM | 13.438 | 19.233 |
| 2μM | 14.817 | 28.44 |
| | None | None |
| 0μM | 4.384999999999994 | 1.986 |
| 1μM | 10.057 | 13.857 |
| 2μM | 18.657 | 31.36 |P<0.0005
P<0.0005
P=0.14
P=0.01
P=0.17
Oxaliplatin
γH2A.X foci / cell
5FU
120h
P<0.0005
P<0.0005
### Chart
| Category | 0μM | 2μM |
|---|---|---|
| 0μM | 9.232 | 20.469 |
| 1μM | 14.145 | 23.939 |
| 2μM | 12.392 | 24.296 |
| | None | None |
| 0μM | 3.061 | 1.323 |
| 1μM | 3.766 | 2.745 |
| 2μM | 4.321999999999996 | 5.319 |P<0.0005
P=0.24
Oxaliplatin
P=0.42
P=0.0007
γH2A.X foci / cell
5FU
SW480
HCT116
Kaneko M, et al.
